# Endodontic flare-ups: comparison of incidence between single and multiple visit procedures in patients attending a Nigerian teaching hospital

**DOI:** 10.1186/1472-6831-4-4

**Published:** 2004-11-26

**Authors:** Adeleke O Oginni, Christopher I Udoye

**Affiliations:** 1 Department of Restorative Dentistry, Faculty of Dentistry, College of Health Sciences, Obafemi Awolowo University, Ile-Ife, Osun State, Nigeria

## Abstract

**Background:**

Until recently the most accepted technique of doing root canal treatment stresses multiple visit procedure. Most schools also concentrated upon teaching the multi-visit concept. However, it has now been reported that the procedure of single visit treatment is advocated by at least 70% of schools in all geographical areas. It was therefore the aims of the present study to find the incidence of post-obturation flare-ups following single and multiple visit endodontic treatment procedures, and to establish the relationship between pre-operative and post-obturation pain in patients referred for endodontic therapy in a Nigerian teaching Hospital.

**Methods:**

Data collected included pulp vitality status, the presence or absence of pre-operative, inter-appointment and post-obturation pain. Pain was recorded as none, slight, or moderate/severe. Flare-ups were defined as either patient's report of pain not controlled with over the counter medication or as increasing swelling. The patients were recalled at three specific post-obturation periods, 1^st^, 7^th ^and 30^th ^day. The presence or absence of pain, or the appropriate degree of pain was recorded for each recall visits and the interval between visits. The compiled data were analysed using chi-square where applicable. P level ≤ 0.05 was taken as significant.

**Results:**

Ten endodontic flare-ups (8.1%) were recorded in the multiple visit group compared to 19 (18.3%) flare-ups for the single visit group, P = 0.02. For both single and multiple visit procedures, there were statistically significant correlations between pre-operative and post-obturation pain (P = 0.002 and P = 0.0004 respectively). Teeth with vital pulps reported the lowest frequency of post-obturation pain (48.8%), while those with nonvital pulps were found to have the highest frequency of post-obturation pain (50.3%), P = 0.9.

**Conclusion:**

The present study reported higher incidences of post-obturation pain and flare-ups following the single visit procedures. However, single visit endodontic therapy has been shown to be a safe and effective alternative to multiple visit treatment, especially in communities where patients default after the first appointment at which pain is relieved.

## Background

Until recently the most accepted technique of doing endodontic treatment stresses multiple visit procedures. Most schools also concentrated upon teaching the multi-visit concept. However, it has now been reported that the procedure of single visit treatment is advocated by at least 70% of schools in all geographical areas [1].

Some of the problems of root canal treatment are post-obturation pain, inter-appointment pain and swelling. Although these in most cases do not last long, but could be a source of embarrassment to the dentist and annoying for the patient, more so if the tooth was symptomless before the commencement of treatment. Literature review revealed varied opinions on the incidence and severity of post-obturation pain. Some authors reported slightly more post-obturation pain following single visit than with multiple visit procedures [2,3]. Others found no significant differences in the post-obturation pain experienced by patients following single or multiple visit treatment procedures [4]. O'Keefe [4] however proposed a correlation between pretreatment pain and post-obturation discomfort. The rate of endodontic flare-ups was reported to be more following multiple visits than for the single visit [5-7], Imura & Zuolo [7] also reported a positive correlation between flare-ups and multiple appointment, retreatment cases, peri-radicular pain prior to treatment and presence of radioluscent lesions. They reported no correlation between post-obturation flare-ups and the status of the pulp. However, Sim [8] reported a significantly higher incidence of flare-ups in necrotic teeth than in vital teeth (p = 0.01). Fox et al. [9] in their study showed that female patients had more post operative pain than did males. Factors of age, bacteriologic status, tooth position and type of filling material showed no clear effect upon post-operative results.

Endodontic treatment in Nigeria is carried out in the department of restorative dentistry of the four dental schools and in few private dental clinics located in major cities, health centres and general hospitals. Previous report revealed that few cases of root canal treatment were undertaken and that root canal treatment was completed in multiple visits, specifically three visits for about half the teeth treated [10]. Reasons for the reported few cases of root canal treatment included patient's preference for extraction, which is a cheaper option (the cost of root canal treatment is about twice that of extraction). Also because most of the patients had to travel a considerable distance for the treatment, they prefer extraction, which is completed in a single visit (except in complicated cases). However, it has been recently observed that the acceptability of endodontic treatment is on the increase among Nigerian patients, with more people desiring to keep their teeth. Despite the desire, they present for treatment late only after the onset of pain. Also some patient do not come back to complete the treatment after the first appointment at which pain is relieved. Hence more dentists are embracing the single visit procedure particularly in the Teaching Hospitals. It was therefore the aims of the present study to find the incidence of post-obturation flare-ups following single and multiple visit endodontic treatment procedures. Establish the relationship between pre-operative and post-obturation pain. Find the incidence and degree of pain at the 1^st^, 7^th^, and 30^th ^post-obturation days, and to compare these results with those reported in previous studies.

## Methods

Consenting patients referred to the department of Restorative Dentistry for root canal therapy within a period of twelve months were randomly assigned for either single visit or multiple visit procedures. For the multiple visit procedures, Patients that defaulted after the first appointment (incomplete treatment) were excluded from the study. For each tooth treated, the clinical factors and conditions existing before, during and after the completion of treatment were recorded. This data included pulp vitality status, the presence or absence of pre-operative pain, post-obturation flare-ups and degree of post-obturation pain. For patients requiring root canal treatment on more than one tooth, the treatment of each tooth was separated by a period of at least four weeks to allow for proper evaluation. The pulp vitality was determined by an electric pulp tester (Parkell pulp vitality tester, Farmingdale, NY 111735) in combination with the presence of pulpal haemorrhage.

The patients were recalled at three specific post-obturation periods, the 1^st^, 7^th ^and 30^th ^day. At each post-obturation recall visit, the patients were interviewed to determine whether or not there were symptoms at the present visit and whether or not there had been symptoms during the interval between the present visit and the previous one. The presence or absence of pain, or the appropriate degree of pain was recorded for each recall visit and the interval between visits. Pain was recorded as none, slight, or moderate/severe. Slight pain was defined as any discomfort no mater how brief in duration that did not require medication and that did not impair masticatory function in any way. Moderate/severe pain was defined as pain requiring medication or other palliative treatment. Impairment of masticatory function (discomfort in chewing) was recorded as moderate/severe pain. Endodontic flare-ups were defined as either patient's report of pain not controlled with over the counter medication and or increasing swelling.

The root canals were obturated with multiple gutta-percha cones and a zinc oxide-eugenol based sealer, using the lateral condensation technique. The compiled data were analysed using chi-square where applicable. P level ≤ 0.05 was taken as significant.

## Results

Two hundred and eighty three (283) teeth in 255 patients were treated in all, given a ratio of 1.11 teeth per patient. Of these 56 were excluded from the study due to non-availability of patients at post-obturation recall visit. These exclusions were randomly distributed between treatment groups, with no differential loss to follow-up (25 from the single visit group, 31 from the multiple visit group). The treatment groups were fairly comparable, with similar distribution of tooth types between treatment groups, Table 1. Two hundred and fourty three (243) were available for check-up on the 1^st ^post-obturation day, of these 107 were completed in single visit and 136 were completed in multiple visit. Eighty-six (86) had vital pulps and 157 had nonvital pulp canal contents. Two hundred and twenty seven (227) reported for check-up on the 7^th ^post-obturation day, of these 104 was completed in single visit and 123 completed in multiple visit. Two hundred and twenty two reported for check-up on the 30^th ^post-obturation day, 102 completed in single visit and 120 in multiple visit.

**Table 1:** Tooth distribution between treatment groups.

	Single visit		Multiple visit	
Tooth types	No	(%)	No	(%)
Maxillary incisors	40	(38.5)	49	(39.8)
Maxillary canines	3	(2.9)	3	(2.4)
Maxillary premolars	20	(19.2)	22	(17.9)
Maxillary molars	6	(5.8)	9	(7.3)
Mandibular incisors	13	(12.5)	12	(9.8)
Mandibular canines	1	(1.0)*	2	(1.6)
Mandibular premolars	9	(8.6)	13	(10.6)
Mandibular molars	12	(11.5)	13	(10.6)
Total	104	(100.0)	123	(100.0)

*Rounded up percentage

Percentage is based on total number in treatment group.

Each interval between visits and subsequent interview were combined and considered as a single post-obturation period. The highest degree of pain reported in either the interval or at the subsequent interview was recorded as the degree of pain for the specific post-obturation period. Ten flare-ups (8.1%), that is patients presenting with pain not controlled by over the counter medication and or increasing swelling, were recorded in the multiple visit group compared to 19 (18.3%) flare-ups for the single visit group. This shows a significant difference (Mantel Haenszel chi-square = 5.18, p = 0.02), Table 2. Of the 107 teeth whose treatments were completed in single visit 67 had pre-operative pain, out of which 50 (74.6%) reported post-obturation pain. Of the 40 teeth with no pre-operative pain, 8 (20%) had post-obturation pain (x^2 ^= 9.04, p = 0.002). For the multiple visit procedures, 88 teeth presented with pre-operative pain out of which 55 (62.5%) reported post-obturation pain. 48 teeth had no pre-operative pain out of which 6 (12.5%) had post-obturation pain (x^2 ^= 12.5, p = 0.0004). These show that for both single and multiple visit procedures, there were statistically significant correlations between pre-operative and post-obturation pain (Table 3).

**Table 2:** Incidence of post obturation flare-ups

Group	Number in study	No flare-ups		Flare-ups present	
		No.	(%)	No.	(%)
Single visit	104	85	(81.7)	19	(18.3)
Multiple visit	123	113	(91.9)	10	(8.1)

Mantel Haenszel Chi square = 5.18, df, = 1, p = 0.02.

**Table 3:** Relationship between pre-operative pain and pain on 1^st ^post obturation day.

Group	No preop. Pain		Postob. Pain		Preop. Pain		Postob. Pain	
	No.	(%)	No.	(%)	No.	(%)	No.	(%)
Single visit(n = 107)	40	(37.4)	8	(20.0)	67	(62.6)	50	(74.6)
							x^2 ^= 9.04, p = 0.002	
Multi Visit(n = 136)	48	(35.3)	6	(12.5)	88	(64.7)	55	(62.5)
							x^2 ^= 12.5, p = 0.0004	

Teeth with vital pulps reported the lowest frequency of pain (48.8%), while those with nonvital pulps were found to have the highest frequency of pain (50.3%), Table 4. The difference was however, not statistically significant (p = 0.90).

**Table 4:** Incidence of pain on 1^st ^post obturation day: Vital and nonvital.

Group	Number in Group	None		Slight		Moderate/severe	
		No	(%)	No	(%)	No	(%)
Vital	86	44	(51.2)	27	(31.4)	15	(17.4)
Nonvital	157	80	(51.0)	52	(33.1)	25	(15.9)

Incidence of pain x^2 ^= 0.02, df = 1, p = 0.90.

Percentage incidence of pain, vital = 48.8.

Percentage incidence of pain, nonvital = 50.3.

The percentages of single visit patients who exhibited slight post-obturation pain on the 1^st ^and 7^th ^days respectively 35.5% and 16.3% were higher than those in the multiple visit group 30.2% and 9.8%. Chi square test indicated no statistically significant differences (Tables 5 &6). The same trend was recorded for moderate/severe pain on the 1^st ^day post-obturation review. The percentage of patient with moderate/severe pain on the 7^th ^day post-obturation was higher for the multiple visits than the single visit group (Table 6). No post-obturation pain persisted to the 30^th ^day.

**Table 5:** Comparison of pain on 1^st ^post obturation day: single and multiple visit.

Group	Number in study	None		Slight		Moderate/severe	
		No.	(%)	No.	(%)	No.	(%)
Single visit	107	49	(45.8)	38	(35.4)	20	(18.7)
Multiple visit	136	75	(55.1)	41	(30.2)	20	(14.7)
Total	243	124	(51.0)	79	(32.5)	40	(16.5)

Incidence of pain: x^2 ^= 1.74, df = 1, p = 0.19, degree of pain: x^2 ^= 2.14, df = 2, p = 0.34.

**Table 6:** Comparison of pain on 7^th ^post obturationday: single and multiple visits.

Group	Number in study	None		Slight		Moderate/severe	
		No.	(%)	No.	(%)	No.	(%)
Single visit	104	87	(83.7)	17	(16.3)	0	(0.0)
Multiple visit	123	109	(88.6)	12	(9.8)	2	(1.6)
Total	227	196	(86.3)	29	(12.8)	2	(0.9)

Incidence of pain: x^2 ^= 0.79, df = 1, p = 0.37.

Teeth with non-vital pulp recorded more post-obturation pain. There was however no significant difference in post-obturation pain between teeth treated (either by the single or multiple visit procedures) whose pulps were non-vital.

## Discussion

Many authorities in the field of endodontics advice against the completion of root canal treatment in single visit in order to prevent post-obturation pain, especially in cases presenting with pre-operative pain [11,12].

In the present study more flare ups occurred in the single visit group (18.3%) than in the multiple visit group (8.1%), showing a disadvantage for single visit treatment at a 95% confidence level, (Table 2). This is in contrast with the findings of Eleazer & Eleazer [6] who reported fewer flare-ups for the single visit group (3.0%) and (8.0%) for the multiple visit group. Other studies also have reported lower incidence figures for endodontic flare-ups [7,13], Walton & Fouad [13] in the United States of America reported an incidence of 3.17%, while Imura & Zuolo [7] in Brazil reported a further lower figure of 1.58%. In Nigeria and possibly in most developing nations, patients do not present for treatment before the onset of severe pain. In most cases they would have tried self prescribed analgesics. These may explain the high incidence of flare-ups reported in the present study, since endodontic flare-ups have been reported to be positively correlated with more severe presenting symptoms and in patients on analgesics [7,13].

Previous studies have shown that there is a strong positive correlation between pre-operative and post-obturation pain [4,14,15]. The present study supports this correlation, in both the single and multiple visit groups there were statistically significant correlation between pre-operative and post-obturation pain, p = 0.002, p = 0.0004 respectively (Table 3). No significant correlation was found between pulp vitality and the reported incidence of post-obturation pain (p = 0.9), Table 4. This finding is in agreement with those of Roan, Dryden & Grimes [16], and Fox et al [9], who reported that whether a tooth pulp was vital or not had little effect on post-obturation pain. It is however in direct conflict with the traditional belief that only vital cases should be considered for single visit endodontics. Although the single visit patients seemed to experience more pain (slight, moderate/severe) than did the multiple visit patients during the first 24 hours, the differences were not statistically significant (Table 5). This finding is supported by that of Pekruhn [17] who also reported no statistically significant difference between the two groups when the total number of pain days was considered, but in contrast with the findings of Roan, Dryden & Grimes [16] that discloses a significant difference in the incidence of post-obturation pain between single and multiple visit.

Despite the high percentages of post-obturation pain reported on the first post-obturation day in both groups (Table 5), seven days after obturation, 83.7% and 88.6% of teeth treated by the single and multiple visit respectively were free of symptoms (Table 6). Also since no post-obturation pain persisted to the 30^th ^day in both groups, these present a strong indication that practitioners should not overreact to early post-obturation symptoms by immediately initiating endodontic retreatment procedures or extraction of the involved tooth. Apart from the reported higher incidence of flare-ups in the single visit group, the post-obturation pain experienced by the patients in both groups compares favourably well with each other. Therefore the higher incidence should not be taken as condemnation for the single visit endodontic therapy, it should however stress the fact that a thorough understanding of the basic endodontic principles is important in considering each case on an individual basis before making a decision as to whether or not it can be completed in one visit [18]. As it is common with all hospital-based studies, the subjects in the present study may not be a true representation of the population. Therefore the ability to generalize the results is weak. However, a careful case selection and adherence to the basic principles of endodontic therapy will reduce the incidence of flare-ups and post-obturation pain and thus enhance a successful outcome.

## Conclusions

The present study reported higher incidences of post-obturation pain and flare-ups following the single visit procedures. However, single visit endodontic therapy has been shown to be a safe and effective alternative to multiple visit treatment, especially in communities where patients default after the first appointment at which pain is relieved.

## Competing interests

The author(s) declare that they have no competing interests.

## Authors' contributions

AOO conceived of the study, participated in its design, performed the statistics, and participated in the final write-up of the manuscript. CIU participated in the design, collected the data, drafted the initial manuscript, and participated in the final write-up of the manuscript.

## Pre-publication history

The pre-publication history for this paper can be accessed here:


